# Supplementary material for: Exploring the Views and Dietary Practices of Older People at Risk of Malnutrition and Their Carers: A Qualitative Study
Source: Nutrients. 2019 Jun 5;11(6):1281. doi: 10.3390/nu11061281 (PMC6627873; doi:10.3390/nu11061281)
Supplement: Supplementary file 1 [file nutrients-11-01281-s001.zip › Supplementary material 1.docx]

**Nutrition in Later Life Study: Draft topic guide for interviews with older people**

- Explain why we are doing the research
- Read and understood information sheet? Opportunity to ask questions
- Audio recording
- Confidentiality and exceptions to this
- Information about types of questions will be asking, no right or wrong answers
- Consent form

| 1. **Older people’s experiences and views on what they currently eat and drink** |
| --- |
| - Can you tell me what you eat in a typical day?   - *Prompt: snacks eaten in between meals, pattern of meal times (eg breakfast / lunch / dinner or as and when hungry), eating alone / with others.*   - *Explore why e.g. How do you decide what makes an okay meal to you?* - Ideally, what would you be eating? - People often talk about having a ‘healthy diet’. What does that mean to you?   - Is there anything that makes it difficult for you to have a ‘healthy diet’? If yes – what? nb explore difficulties eating eg dentures. Financial constraints. - What do you drink in a typical day?   - *Prompt: explore different drinks consumed including water, tea/coffee, milk, juices, alcohol etc* - What do you think is a healthy amount of fluid to drink each day? - At times you may need to increase the amount you drink, eg if you’re feeling unwell or its very hot. How do you go about making sure you drink enough at times like this? - Where do you tend to eat your meals? |
| 1. **Meal preparation and shopping** |
| - How do you find preparing a meal?   - *Explore if willing to spend time in meal preparation* - Is anyone else involved in planning your meals? If yes, who? - What help do you need to prepare a meal? - How do you go about shopping for food?   - *Prompt: re frozen food, ready-made meals etc. Explore access / transport including public transport journey, also views on shopping eg chore/social* - How do you decide what you are going to buy? |
| 1. **Nutritional changes in later life** |
| - It is common that people notice changes to their appetite as they become older. Have you noticed any changes to your eating habits recently, compared to when you were younger?   - If yes, can you think of any reasons for this? - How is your appetite?   - If appetite has changed, enquire whether they have made any changes to how they eat eg frequency, snacks. - How are your energy levels? Does this vary during the day?   - If energy levels have changed, ask whether this bothers them? If yes, how? - Have you noticed any changes in your weight recently?   - If lost weight enquire if intentional, any changes to quantity/portion size consumed.   - (If unintentional weight loss ask if has seen GP about their weight) |
| 1. **Experience with use of current services**  - Have you ever received advice about your diet?   - If yes - where, what type of advice, what did you think of it? - Have you ever spoken to your primary care team about their diet, weight?   - If yes – what did you think of the discussion and their advice?   - If no – would you consider it and why? - How do you feel about your weight? |
| 1. **Older people’s views around changing their diet and sustainability** |
| - If a Dr or nurse recommended that you should eat more / put on some weight, what would you think of that?   - Would you make any changes yourself? If yes – what and how? If no – why?   - What support do think could help you? - How would you feel about changing the amount you drink if this was advised? - What would help you to maintain these changes long term?   - *Probe: re ability to do this alone/support needed, motivation* - If difficult to talk about personal experiences ask about peers. |
| 1. **Support to help older people who have lost weight/reduced appetite/reduced fluid intake** |
| We are designing a new service to support those who have noticed changes to their appetite/energy levels about how to maximise protein and calorie consumption. We would like you views on what such a service or support could look like and how best to deliver it.   - What would you think about using a service like this? - What type of support would you like to receive about your diet or weight?   - Reading material/leaflets, 1-to-1, group based sessions - Who would be the best person to provide that type of support? - Or who would you be willing to accept advice from?   - Prompt re health professional or non-health trained, GPs, PNs, HCAs   **Close, thanks and voucher** |

Participant ID:

**About You**

1. What is your gender?

Male

Female

1. What is your age group?

75-79

80-84

85-89

90+

1. What is your ethnic group?

White

1. English/Welsh/Scottish/Northern Irish/British
2. Irish
3. Gypsy or Irish Traveller
4. Any other white background: …………………………

Mixed/multiple ethnic groups

1. White and black Caribbean
2. White and black African
3. White and Asian
4. Any other mixed/multiple ethnic backgrounds:……………………………………………

Asian/Asian British

1. Indian
2. Pakistani
3. Bangladeshi
4. Chinese
5. Any other Asian background:………………………….

Black/African/Black Caribbean/Black British

1. African
2. Caribbean
3. Any other black/African/Caribbean background:……………………………………………..

Other ethnic group

1. Arab
2. Any other ethnic group:………………………………..
3. What are your current living arrangements?
4. I live alone
5. I live with my spouse/partner
6. I live with another family members/other family members
7. I live with a friend/someone else
8. Other (please describe) ……………………………….
9. What is your current marital/civil partnership status?
10. Single, never married/civil partnership
11. Co-habiting with partner
12. Married or in a civil partnership
13. Separated, but still legally married/in a civil partnership
14. Divorced
15. Widowed
16. Which of the following best describes your current housing?
17. Owner-occupied
18. Council rented
19. Housing Association rented
20. Social housing rented
21. Private rented
22. Sheltered housing (specify whether council/housing association/social housing/private)
23. Other (please specify) …………………………………
24. At what age did you complete your education in school or college?
25. Before the age of 15 years
26. At the age of 15 or 16 years
27. Between the ages of 17 and 20 years
28. After the age of 21 years
29. What type of pension do you receive (please tick all that apply)?
30. State pension
31. Employer pension
32. Private pension
33. Not applicable
34. Do you receive any of the following benefits (please tick all that apply)?
35. Pension credit
36. Disability Living Allowance or Attendance Allowance
37. Housing benefit
38. Council Tax benefit

Any other benefits (please specify)……………………….
