# Supplementary material for: Exploring the Views and Dietary Practices of Older People at Risk of Malnutrition and Their Carers: A Qualitative Study
Source: Nutrients. 2019 Jun 5;11(6):1281. doi: 10.3390/nu11061281 (PMC6627873; doi:10.3390/nu11061281)
Supplement: Supplementary file 1 [file nutrients-11-01281-s001.zip › Supplementary material 2.docx]

**Nutrition in Later Life Study**

**Draft topic guide for interviews with carers or support givers of older people**

- **Read and understood information sheet?**
- **Opportunity to ask questions**
- **Audio recording**
- **Confidentiality and exceptions to this**
- **Information about types of questions will be asking, no right or wrong answers**
- **Consent form**

1. **Carers’ views on what older people currently eat and drink**

- What does the older person you care for eat in a typical day?
  - *Prompt: snacks eaten in between meals, pattern of meal times (eg breakfast / lunch / dinner or as and when hungry), eating alone / with others.*
- *Explore why e.g. How do you decide what makes an okay meal for the person you are supporting?*
- Ideally, what would they be eating?/Would they be eating anything different?
- What do you think of what they currently eat?
- What do you think is a ‘healthy diet’ for an older person?
  - Is there anything that makes it difficult for them to have a ‘healthy diet’? If yes – what? nb explore difficulties eating eg dentures. Financial constraints.
- What does the older person you care for drink in a typical day?
  - *Prompt: explore different drinks consumed including water, tea/coffee, milk, juices, alcohol etc*
- What do you think is a healthy amount of fluid for an older person to drink each day?
- At times older people may need to increase the amount they drink. How do you go about making sure they drink enough at times like this?

1. **Meal preparation and shopping**

- What is your role in assisting the older person preparing their meal (planning their meals, shopping, cooking)?
- Are there other people involved in preparing their meals? In what way?
- Do you help [x] with shopping for food?
  - *Prompt: re frozen food, ready-made meals etc. Explore access / transport including public transport journey, also views on shopping eg chore/social*
- If yes, how do you decide what you are going to buy?
- Where does the older person tend to eat their meals?

1. **Nutritional changes in later life**

- Have you noticed any changes to [x]’s eating habits as they have become older?
- If yes, can you think of any reasons for this?
- How is the older person’s appetite?
  - If appetite has changed, enquire whether they have made any changes to how they eat eg frequency, snacks.
- How are their energy levels?
  - If energy levels have changed, ask whether this bothers them? If yes, how?
- Have you noticed any changes in their weight?
  - If lost weight enquire if intentional, any changes to quantity/portion size consumed.

(If unintentional weight loss ask if has seen GP about their weight)

Has the older person ever been told they are underweight?

1. **Carers’ views around older people changing their diet and sustainability**

- How would you feel about changing what the older person you care for eats?
- How would you feel about the older person changing the amount they drink if this was advised?
- What would motivate them to do so?
- Would they feel able of doing it?
- What would help them to maintain these changes long term?

1. **Experience with use of current services**

- Have you ever received advice by somebody else regarding the older person’s diet? (if yes where, what type of advice, what did you think?)

1. **Design of a new service to help older people who have lost weight/reduced appetite/reduced fluid intake**

We are designing a new service to support those who have noticed changes to their appetite/energy levels about how to maximise protein and calorie consumption. We would like you views on what such a service or support could look like and how best to deliver it.

- What would you think about using a service like this?
- What type of support would you like to receive from a service?
  - Reading material/leaflets, 1-to-1, group based sessions
- Who would be the best person to provide that type of support?
- Or who would you be willing to accept advice from?
- Prompt re health professional or non-health trained, GPs, PNs, HCAs
- Where would you prefer this type of service to be based?

Participant ID:

**About You**

1. What is your gender?

Male

Female

1. What is your age group?

18-29

30-39

40-49

50-59

60-69

70-79

80-89

90+

1. What is your ethnic group?

White

1. English/Welsh/Scottish/Northern Irish/British
2. Irish
3. Gypsy or Irish Traveller
4. Any other white background: …………………………

Mixed/multiple ethnic groups

1. White and black Caribbean
2. White and black African
3. White and Asian
4. Any other mixed/multiple ethnic backgrounds:……………………………………………

Asian/Asian British

1. Indian
2. Pakistani
3. Bangladeshi
4. Chinese
5. Any other Asian background:………………………….

Black/African/Black Caribbean/Black British

1. African
2. Caribbean
3. Any other black/African/Caribbean background:……………………………………………..

Other ethnic group

1. Arab
2. Any other ethnic group:………………………………..
3. What is your relationship to the person you are helping with food shopping/meal preparation? ………………………………………………………………………………………………………………………………………………………………………………………………………………………........................................
